# Antisera Produced Using an *E. coli*-Expressed SARS-CoV-2 RBD and Complemented with a Minimal Dose of Mammalian-Cell-Expressed S1 Subunit of the Spike Protein Exhibits Improved Neutralization

**DOI:** 10.3390/ijms241310583

**Published:** 2023-06-24

**Authors:** Takahiro Yoshizue, Subbaian Brindha, Rawiwan Wongnak, Hitoshi Takemae, Mami Oba, Tetsuya Mizutani, Yutaka Kuroda

**Affiliations:** 1 Department of Biotechnology and Life Science, Faculty of Engineering, Tokyo University of Agriculture and Technology, 2-24-16 Nakamachi, Koganei-shi 184-8588, Japan; s214129v@st.go.tuat.ac.jp (T.Y.); s218131w@st.go.tuat.ac.jp (R.W.); 2 Institute of Global Innovation Research, Tokyo University of Agriculture and Technology, 3-8-1 Harumi-cho, Fuchu-shi 183-8538, Japan; fy7210@go.tuat.ac.jp (H.T.); mamioba@go.tuat.ac.jp (M.O.); tmizutan@cc.tuat.ac.jp (T.M.); 3 Center for Infectious Disease Epidemiology and Prevention Research, Tokyo University of Agriculture and Technology, 3-5-8 Saiwai-Cho, Fuchu-shi 183-8509, Japan

**Keywords:** subunit antigen, *Escherichia coli* expression, mammalian cell expression, immunogenicity, pseudovirus neutralization

## Abstract

*E. coli*-expressed proteins could provide a rapid, cost-effective, and safe antigen for subunit vaccines, provided we can produce them in a properly folded form inducing neutralizing antibodies. Here, we use an *E. coli*-expressed SARS-CoV-2 receptor-binding domain (RBD) of the spike protein as a model to examine whether it yields neutralizing antisera with effects comparable to those generated by the S1 subunit of the spike protein (S1 or S1 subunit, thereafter) expressed in mammalian cells. We immunized 5-week-old Jcl-ICR female mice by injecting RBD (30 µg) and S1 subunit (5 µg) according to four schemes: two injections 8 weeks apart with RBD (RBD/RBD), two injections with S1 (S1/S1), one injection with RBD, and the second one with S1 (RBD/S1), and vice versa (S1/RBD). Ten weeks after the first injection (two weeks after the second injection), all combinations induced a strong immune response with IgG titer > 10^5^ (S1/RBD < S1/S1 < RBD/S1 < RBD/RBD). In addition, the neutralization effect of the antisera ranked as S1/RBD~RBD/S1 (80%) > S1/S1 (56%) > RBD/RBD (42%). These results indicate that two injections with *E. coli*-expressed RBD, or mammalian-cell-produced spike S1 subunit alone, can provide some protection against SARS-CoV-2, but a mixed injection scheme yields significantly higher protection.

## 1. Introduction

Despite the recession of the COVID-19 pandemic, the causative agent of severe acute respiratory syndrome coronavirus 2 (SARS-CoV-2) [1] remains latent in the global population. SARS-CoV-2 is a single-stranded, positive-sense RNA virus and belongs to the Coronaviridae family [2]. Its genome encodes four structural proteins: the spike (S), envelope, membrane, and nucleocapsid proteins [2]. The Spike protein of the SARS-CoV-2 virus (1273 amino acids (aa)) is a homotrimeric transmembrane (TM) protein and contains an S1 subunit (aa: 14–685) and an S2 subunit (aa: 686–1273) [3]. The S1 subunit, responsible for binding the virus to host cell receptors, is composed of the N-terminal domain (NTD; aa: 18–305) and the C-terminal receptor-binding domain (RBD; aa: 319–541) [3]. The receptor-binding domain (RBD) is composed of two sub-domains including a core sub-domain, composed of a β-sheet with five anti-parallel strands (β1, β2, β3, β4, and β7) in the inner side of the Spike protein, and a receptor-binding motif (RBM) from the outer side that extends from the core sub-domain and consists of β5 and β6 strands [4,5]. The S2 subunit of the Spike protein mediates the fusion of the viral membrane with the host cell membrane allowing viral entry into the target cell.

Virus binding occurs through RBD (and thus RBM) binding to ACE2 [3], a membrane-bound zinc-containing enzyme angiotensin-converting enzyme 2. ACE2 is expressed in many tissues including lungs, arteries, heart, kidneys, and intestines, and is the main receptor for virus attachment to target cells [6]. The spike protein and its RBD domain are thus a prime target for anti-SARS-CoV-2 vaccines [7]. The spike protein is the antigen used in all of the current vaccines [8], including the mRNA vaccines (Moderna and Pfizer-BioNTech), the replication-incompetent adenovirus vaccine (Johnson & Johnson and AstraZeneca), and the recombinant Spike protein (Novavax and Sanofi) [9].

A safe and cost-effective vaccine thus remains a practical way to prepare against future outbreaks. In addition to the widely used and famed anti-SARS-CoV-2 mRNA vaccine, vector-based, inactivated virus, subunit, and DNA vaccines [10] are currently being approved or used against SARS-CoV-2. Like all vaccines, potential disadvantages and possible side effects are reported for each modality. For instance, in inactivated and live-attenuated virus-based vaccines, there is a possibility for incomplete inactivation of viruses or the existence of recovering virulence, resulting in significant safety concerns [11], and this may induce antibody-dependent enhancement (ADE) effect, as in the case of SARS-CoV infection [12]. Similarly, some viral-vectored vaccines can induce anti-vector immunity. Successful mRNA/nucleic-acid-based vaccines require high manufacturing expertise and complex storage and distribution cold chain [13]. The other challenges are the emergence of variants evading the protection of existing vaccines and demanding repeated vaccinations. Subunit antigens have the potential to deliver a cost-effective and robust alternative to other vaccine modalities ([14] and references therein).

In addition, its rapid rate of mutation has resulted in highly contagious viral strains evading the protection provided by existing vaccines, which has led to a steady rise in infection rates. Developing vaccines for novel strains can be time-consuming, especially for traditional vaccines that use the whole virus. The development of mRNA and subunit vaccines against novel variants was substantially shorter as they take advantage of the existing vaccine as a template wherein the mutations are inserted. 

SARS-CoV-2 RBD contains 9 cysteines that form 4 disulfide bonds, stabilizing the native structure [9]. Their expression in a eukaryotic system, having the molecular machinery to refold proteins into their native structure by reshuffling aberrant SS bonds, would be a natural choice for producing SS-bonded antigen proteins [15]. However, protein production in mammalian cells is labor-intensive and time-consuming [16]; prokaryotic expression systems like *E. coli* can offer a convenient production system, provided the SS bond and protein refolding issues are overcome [16,17,18,19].

We have previously shown that RBD produced in *E. coli* can be refolded into a native-like structure with binding activity to ACE2 [17]. The *E. coli*-produced RBD proteins elicited a strong anti-RBD IgG response in mice models, which produced neutralizing antisera. Here, we ask how the immune response generated by the *E. coli*-produced RBD compares to that induced by the S1 subunit of the Spike protein produced in mammalian cells, serving as a model for a subunit SARS-CoV-2 vaccine. In addition, we investigate whether optimizing RBD’s formulations can improve the antisera’s neutralization activity. Interestingly, we found that one injection with RBD, supplemented with another injection containing the mammalian cell-produced S1 subunit, yields a significantly stronger neutralization effect than two doses of the S1 subunit protein. 

## 2. Results and Discussion

### 2.1. Production and Physico-Chemical Characterization of E. coli Produced RBD

**Figure 1:**
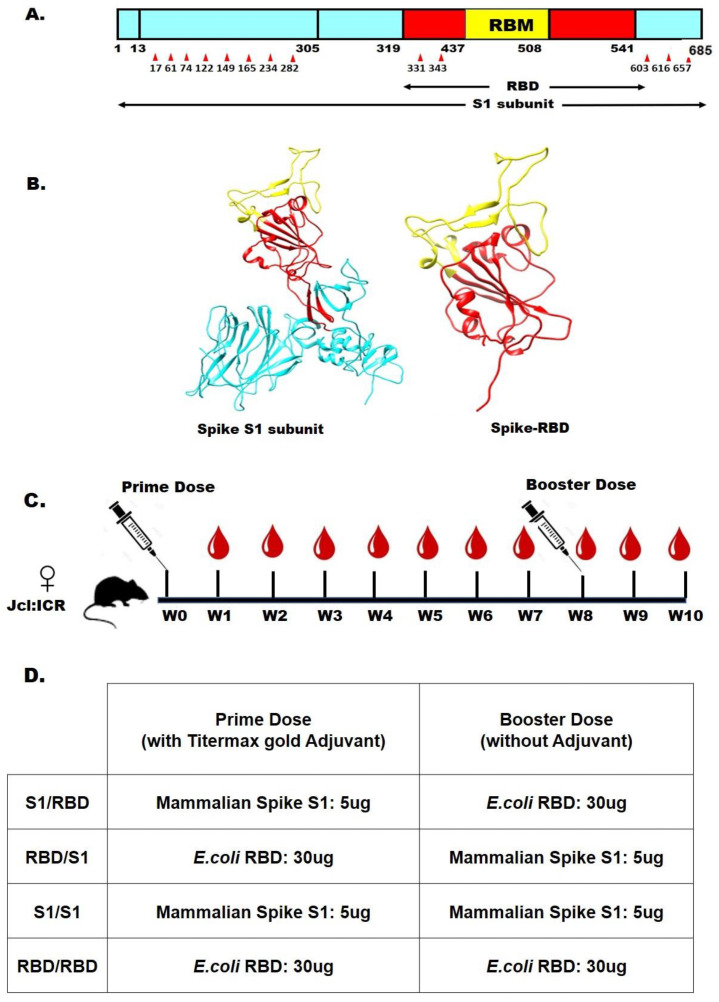
(**A**) Schematic domain representation of the S1 subunit and RBD, receptor-binding domain, on the SARS-CoV-2 Spike protein; red triangles indicate the glycosylation sites. (**B**) Ribbon model of S1 subunit (left) and RBD (right). The pictures were generated using Swiss model [23] with PDB ID (8DV2). (**C**) Mice Immunization scheme and schedule. (**D**) Mice immunization groups and their antigen formulation.

In this study, we used RBD produced in SHuffle T7 *E. coli* (Figure 1). For certain viruses (e.g., respiratory syncytial virus, RSV), folded viral surface antigens [20] or immunogens mimicking the conformation of the native and folded antigen [21] are required for inducing neutralizing antibodies. Similarly, high neutralization of SARS-CoV-2 needs immunization with folded RBD [22], and we thus confirmed the conformational and biophysical properties of the freshly purified RBD according to the protocols reported in [17].

**Figure 2:**
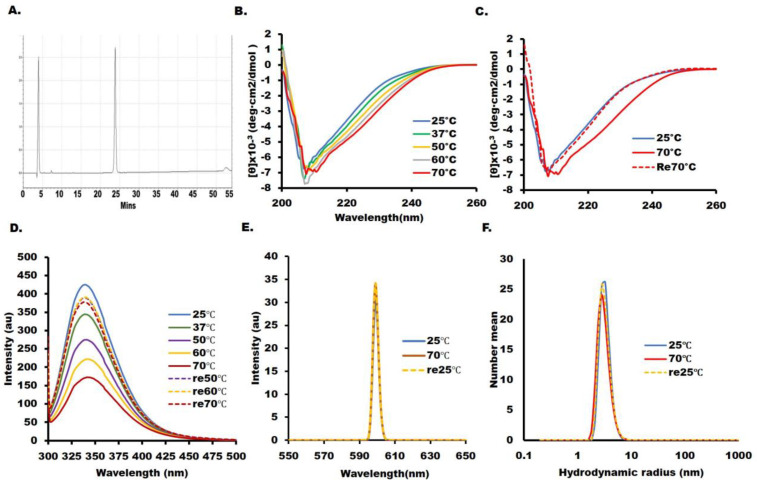
Biophysical and spectroscopic characterization. All spectroscopic measurements were performed at a protein concentration of 0.3 mg/mL in 10 mM Hepes buffer, pH 6.0. Characterization of the secondary structures. The conditions are the same as in [10], but new measurements were performed to confirm reproducibility. (**A**) RP−HPLC purification: the elution profile of recombinant SARS−CoV−2 RBD shows the presence of a single peak. (**B**) The secondary structures were analyzed using the far−UV CD region (200−260 nm). (**C**) Reversibility of denaturation by CD at 70 °C to 25 °C. (**D**) Characterization of the tertiary structures: tryptophan fluorescence intensity used a wavelength of 295 nm for excitation and 345 nm for emission. (**E**) SARS−CoV−2 RBD particle sizes are measured by static light scattering (SLS) and (**F**) dynamic light scattering (DLS). Line symbols are explained within the panels.

Hence, the conformational integrity of the RBD was assessed by circular dichroism and tryptophan fluorescence spectroscopy under the same conditions as in our previous report [17]. The far UV-CD spectrum was measured at pH 6 (Figure 2B,C), and the secondary structure content computed from the far UV-CD spectrum using BestSel was 14.0% (19.07%) α-helix and 28.9% (29%) β-sheet, in good agreement with the crystal structure PDB: 6M0J (values in the parentheses indicate the X-ray structure values) and with our previously published results [17]. The tryptophan fluorescence intensity of SARS-CoV-2-RBD at 25 °C, with a maximum emission of 339 nm, exhibited reversibility upon heating up to 70 °C (Figure 2D). The extent of solubility and particle size of the RBD was assessed by SLS and DLS, respectively. The SLS intensity remained significantly low, and the Rh was 2.19 nm at pH 6.0, consistent with a monomeric RBD (Figure 2E). SLS indicated the absence of aggregation.

### 2.2. Immune Response

**Figure 3:**
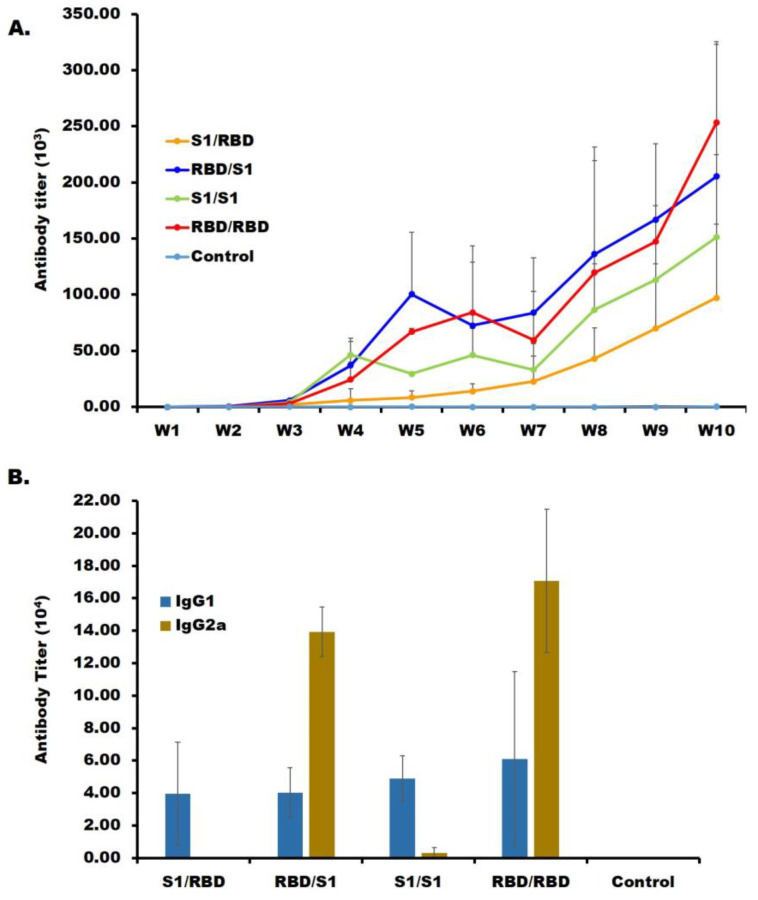
Anti-SARS-CoV-2 RBD antisera (IgG) titer assayed by ELISA; IgG detection by ELISA was performed using the tail bleeding (TB) antisera. W indicates the tail bleeding week. (**A**) Each line shows the average IgG titer of the mice in each group. (**B**) Average IgG sub-class (IgG1, IgG2a) determination from each immunization group with tail bleeding 10 antisera. The results are indicated as mean ± standard error (SE).

All the immunization groups elicited robust immune responses, lasting for 13 weeks (Figure 3A and up to 13 W; S1, S2). In all the groups, the IgG titers increased significantly two weeks after the second injection (by 2 to 2.5 folds; Figure 3A). Two injections with *E. coli*-produced RBD (RBD/RBD group) induced an immune response more robust than two injections with mammalian−cell−produced S1 subunit protein (S1/S1 group), which was unexpected (Figure 3A). This observation is tentatively explained by *E. coli* RBD having a flexible structure, which would yield a better antigen presentation than the mammalian−cell−produced S1 subunit protein. Alternatively, one can speculate that some epitopes may be glycan−shielded in the S1 subunit protein [24], whereas the *E. coli* RBD presents epitopes to the immune system due to the absence of glycosylation (there are two potential sites in RBD) [10]. In addition, the RBD/S1 group had an IgG titer of ~200,000, and the S1/RBD group had the lowest IgG titer with around ~97,000 (Figure 3A). The immunization groups RBD/S1 and RBD/RBD elicited a cell-mediated Th1 immune response (Figure 3B), as indicated by an IgG2a titer higher than IgG1. This observation corroborates our previous results [17], wherein the RBD with TiterMax gold adjuvant shifted the immune response towards the Th1 response. However, mice in the S1/RBD and S1/S1 groups exhibited a humoral Th2 immune response, as shown by an IgG1 subclass titer higher than that of IgG2 (Figure 3B). Finally, we note that mice injected at least once with *E. coli* RBD had persistently higher titer (by an average of >90,000) than those injected twice with S1 only.

### 2.3. Neutralization Assay

We assessed the neutralization effect of the antisera using a pseudovirus neutralization assay (see Section 3 for details). Here, the SARS-CoV-2 (D614G) pseudovirus expresses the S1 subunit, which mediates entry into the host cells by binding to the human angiotensin-converting enzyme 2 (ACE2). Neutralizing antisera contain antibodies that bind to regions of the Spike protein, hampering the S subunit’s interaction with ACE2. Upon infection, the pseudoviruses release RNA into the cells, which are reversely transcribed, and integrated into the gene, encoding the spike protein into the target cell’s genome. Hence, the gene copy number of the spike protein provides a measure of the viral infection.

**Figure 4:**
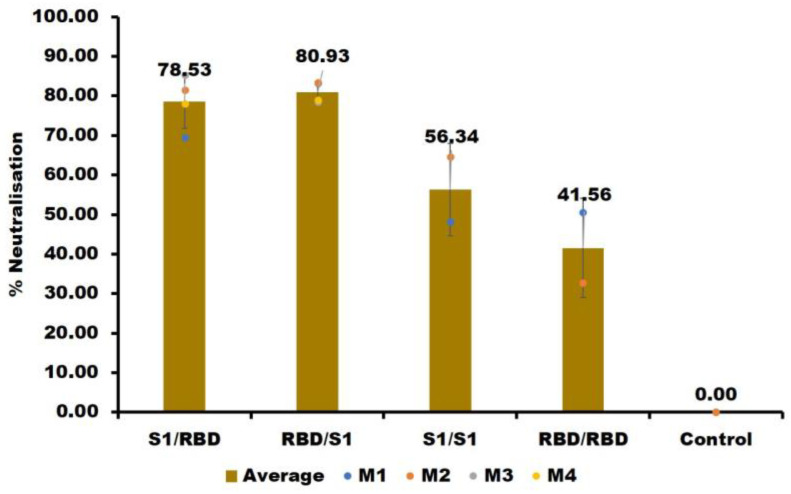
Vero E6/TMPRSS2 cells were transduced with a SARS-CoV-2 S (D614G) pseudotyped lentivirus in the presence of mouse antisera. Twenty-four hours later, the cells were collected, and RNA was extracted. The copy number of the S gene (codon-optimized) was examined to determine the percentage of neutralization. Data are means and standard deviations of triplicates from one representative experiment. Dots represent the individual mice’s % inhibition, and the bar represents the mean values of % inhibition. The statistical significance of the neutralization titers was analyzed by TukeyHSD analysis for multiple comparison tests. There was no significant difference between S1/RBD and RBD/S1, S1/S1, and RBD/RBD, whereas differences between the other combinations are significant (*p* < 0.05).

The 10th-week antisera were used for the neutralization assay. The antisera from groups 3 and 4 neutralized SARS-CoV-2 pseudoviruses with an efficacy of 56.34% (S1/S1) and 41.55% (RBD/RBD), respectively (Figure 4, Table S1). We hypothesize that the increased neutralization of the S1/S1 group originates from epitopes located in S1 (Figure 1A), but outside the RBD, including those in the N terminal domains [25]. The lesser neutralization might also arise from the absence of glycosylation in *E. coli*-produced RBD. Finally, the neutralization by antisera produced by combining mammalian-cell-produced S1 subunit protein and *E. coli*-produced RBD (S1/RBD and RBD/S1) was substantially higher than those of RBD/RBD and S1/S1 antisera, in line with our motivation for starting the experiments.

Two limitations of our current study are the limited follow-up time and the role of the S2 region of the Spike protein. Firstly, the induction of long-lasting B cell and T cell immune memory could be considered in future studies. Secondly, the S2 subunit elicits the production of anti-SARS-CoV-2 specific antibodies [26], but it is also reported that S2-produced antisera are weakly or non-neutralizing [27]. In addition, antibodies isolated from SARS-CoV-2 patients binding the S2 fusion peptide reportedly neutralize alpha and beta coronaviruses [28]. Thus the role of the S2 subunit in producing neutralizing anti-sera remains debatable. Thus, a live virus neutralization assay is worth considering for developing an RBD/S1-based subunit vaccine.

## 3. Material and Method

### 3.1. Protein Expression, Purification

RBD (UniProt ID P0DTC2) with Cys538Ala was expressed in SHuffle T7 *E. coli* cells (New England Biolabs, Ipswich, MA, USA), using a pET15b expression vector in LB medium containing 50 μg/mL ampicillin as previously reported [10]. The protein was purified by using denaturing nickel–nitrilotriacetic acid (Ni-NTA) (Wako, Tokyo, Japan) chromatography and reverse-phase (RP) high-performance liquid chromatography, lyophilized and stored at −30 °C until use [17].

### 3.2. Spectroscopic and Binding Affinity Measurements

The measurements were newly performed to confirm the reproducibility of our observations under the immunization condition, which was 10 mM Hepes buffer and pH 6.0 at a protein concentration of 0.3 mg/mL. The measurements were carried out, in essence, as reported previously [17].

We measured the biophysical properties of the proteins in 10 mM Hepes buffer, at pH 6.0 and at a protein concentration of 0.3 mg/mL, which is the concentration we used for the immunogenic studies. Stock solutions were prepared by dissolving the protein in MQ. The stock solutions were centrifuged at 20,000× *g* for 20 min at 4 °C and filtered with a 0.20 μm membrane filter (MDI Membrane Technology, India) to remove any aggregates. The final samples were prepared by diluting the stock solutions to a final protein concentration of 0.3 mg/mL in 10 mM Hepes buffer (pH 6.0). Far-UV circular dichroism (CD): CD was measured using a 2 mm-path-length quartz cuvette in a continuous scanning mode. For each measurement, three scans were accumulated from 200 to 260 nm wavelengths at temperatures from 25 °C to 70 °C with 10 °C increments. Reversibility was assessed by measuring the spectrum after cooling the sample to 25 °C. The secondary structure content was calculated using BeStSel. Tryptophan fluorescence: Trp fluorescence spectra with λex at 295 nm, respectively, were measured on an FP-8500 spectrofluorometer (JASCO, Tokyo, Japan) using a quartz cuvette with a 3 mm optical path length. The sample’s temperature increased from 25 °C to 70 °C and decreased back to 25 °C to assess reversibility. Light scattering: Dynamic light scattering (DLS) measurements were performed at 25 °C, 70 °C, and reverse 25 °C for pH 8.0, using a polystyrene cuvette with a Zeta-nanosizer (Malvern, UK). The hydrodynamic radius [*R*_h_] was determined from the size distribution using the Stokes–Einstein equation and averaged over three individual measurements. Static light scattering (SLS) measurements were performed under the same conditions at a wavelength of 600 nm with an FP-8500 spectrofluorometer (JASCO, Tokyo, Japan) using a quartz micro-cuvette with an optical path length of 3 mm. The RBD binding affinity with hACE2 (Bioworld Tech, MN, USA) was measured using an OCTET-N1 Biomolecular Interaction Analyzer (Satorius, Inc., USA). A 5 μg/mL concentration of SARS-CoV-2 RBD was immobilized on the biosensor surface for 300 s. The baseline interference phase was obtained by measurements taken for 60 s in kinetics buffer (KB: 1x Hepes and 0.02% Tween-20), and then the sensors were subjected to association phase immersion for 400 s in wells containing recombinant hACE2 diluted in HEPES buffer. Then, the sensors were immersed in KB for as long as 400 s in the dissociation step. The RBD binding affinities for ACE2 were calculated from global fitting with a 1:1 Langmuir binding model.

### 3.3. Mice Immunization

All mouse experiments were performed according to the animal ethics guidelines and protocols set by the Tokyo University of Agriculture and Technology and the Japanese governmental regulations on animal experimentation. Five-week-old female Jcl: ICR mice (CLEA Japan Inc., Shizuoka, Japan) were immunized by subcutaneous injection of the first dose into the loose skin of the neck, and a second dose was administered intraperitoneally after eight weeks. Antigens administered subcutaneously stay localized, whereas the antigens administrated intraperitoneally disperse rapidly in the body. Formulations with adjuvants may induce pain/discomfort to the mice, and we thus administrated the first dose subcutaneously as it contains an adjuvant. For the first dose, the antigens RBD or the S1 subunit protein (Invivogen, San Diego, CA, USA) were dissolved in 10 mM Hepes buffer, pH 7.4, and supplemented with an equal volume of TiterMax Gold adjuvant (Sigma–Aldrich, St. Louis, MO, USA) (100 µL protein plus 100 µL adjuvant for a total of 200 µL/dose/mice). No adjuvant was used in the second dose (100 µL of total volume). The concentrations of 0.3 mg/mL of *E. coli* RBD and 0.05 mg/mL of mammalian-cell-produced S1 protein were used in formulations. Mice were separated into 4 groups (*n* = 4, 4, 2, 2 for groups 1, 2, 3, 4, respectively) and immunized according to the scheme in Figure 1D. Serum samples were collected every week by tail bleeding to monitor the immune response. The blood sample was centrifuged at 1200× *g* for 15 min and stored at −20 °C for ELISA and neutralization assays. The mice were euthanized after 13 weeks, and blood samples were collected from the heart. The antisera were preserved at −30 °C in 50 µL until use.

### 3.4. Measurement of IgG Titer

The antisera’s IgG, IgG1, and IgG2a levels were assessed individually by ELISA, using 2.5 µg of *E. coli* RBD as a coating antigen, in a 96-well 4HBX Immulon (Thermo Fisher Scientific, Waltham, MA, USA) plate at 4 °C overnight. Namely, a high IgG1/IgG2 ratio indicates a Th2-type humoral immune response, whereas a low one indicates a Th1-type cellular immune response. SARS-CoV-2 antisera were applied to the ELISA plates at an initial dilution of 1:1000 for tail-bleed samples. Plates were then incubated at 37 °C for 1 h. After washing the plates thrice with PBS-0.05% Tween-20, each well was filled with 100 μL of anti-mouse IgG HRP conjugate (Thermo Fisher Scientific, Waltham, MA, USA) at a 1:3000 dilution (or its subclasses-IgG1, 1:25,000 dilution/IgG2a, 1:3000 dilution) in 0.1% BSA-PBS-Tween-20 and incubated at 37 °C for 1 h. As a substrate, OPD (o-phenylenediamine dihydrochloride) was added. Color intensities were measured at 492 nm using a microplate reader (SH9000 Lab, Hitachi High-Tech Science Co., Tokyo, Japan) immediately after stopping the reaction with 1 N sulfuric acid (50 µL/well) [29,30,31]. Antibody titers were calculated from the power fitting of OD 492 nm versus the reciprocal of the antisera dilution, using a cutoff of OD 492 nm = 0.1 above the background value. The values were averaged over the number of mice (*n*) in the respective groups.

### 3.5. Pseudovirus-Based Neutralization Assay

The 10-week antisera were used to evaluate the neutralizing efficacy. The neutralization assay was performed according to our reported protocol [17]. Antisera were heat-inactivated at 56 °C for 30 min before use. Neutralizing antibodies were detected using pseudoviruses. The SARS-CoV-2 variant pseudovirus was prepared according to the protocol of Lenti-Pac SARS-CoV-2 full-length spike protein-pseudotyped (D614G) lentivirus packaging kit (GeneCopoeia, Rockville, Maryland, USA). Briefly, 293T cells (2.5 × 10^5^ cells) were seeded onto a 6-well culture plate in DMEM/5%FBS 2 days before transfection. The cells were then transfected with 5 μg of Pseudo type packaging mix using EndoFectin Lenti transfection reagent. At 16 h post-transfection, the culture medium was replaced with 2 mL of DMEM/2%FBS, and TiterBoost Reagent was added to the medium. At 48 h post-transfection, the supernatant containing SARS-CoV-2variant (D614G) pseudovirus was collected and stored at −80 °C until use. For the neutralization assay, Vero E6/TMPRSS2 cells were seeded onto a 24-well culture plate in EMEM/5%FBS 24 h before assay. The D614G pseudovirus (135 μL) was mixed with 15 μL of heat-inactivated mice antiserum and incubated at 37 °C for 1 h. The virus–antiserum mixture was added to the cells in EMEM/5% FBS containing polybrene (final: 10 μg/mL). After 24 h incubation at 37 °C in a 5% CO_2_ incubator, the cells from each well were harvested separately, and RNA isolation was performed using a Qiagen RNAeasy kit (Hilden, Germany) according to the manufacturer’s protocol. For the qPCR assay, one hundred ng of total RNA was amplified using One Step PrimeScript RT-PCR Kit (Takara Bio, Otsu, Japan) as described before [32]. Primers and probes were designed from the codon-optimized S gene sequence of the D614G pseudovirus. The sequences were as follows: primer F: 5′-GCAAGATCGCCGACTACAA-5′, primer R: 5′-TTGCCTCCAACTTTCGAATCTA-3′, and probe: 5′-56-FAM/TACAAGCTG/ZEN/CCTGACGACTTCACC/3IABkFQ/-3′. The thermal cycling condition was 45 °C for 5 min and 95 °C for 30 s, followed by 40 cycles of 95 °C for 10 s, 55 °C for 20 s, and 72 °C for 20 s. Fluorescence signal data were analyzed using an automatic quantification algorithm in LightCycler Nano Software 1.1 (Roche Diagnostics, Tokyo, Japan).

### 3.6. Statistical Analysis

Statistical analyses were performed using the EZR software [33]. The statistical significance of the neutralization titers was analyzed by TukeyHSD analysis for multiple comparison tests with *p* < 0.05. Data are presented as the mean ± the standard deviation (SD).

## 4. Conclusions

Our results suggest that combining antigens from different production platforms and with different domains act synergistically and improves the neutralization effects. In particular, it was unanticipated that combining one injection with the mammalian-cell-produced antigen and one with the *E. coli*-produced RBD yields a higher neutralization than two injections with the mammalian-cell-produced S1 protein. The synergetic effect of combining antigens from different expression systems and spanning different domains/subunits is worth further examination as it might result in a versatile and cost-effective subunit vaccine.

## Data Availability

Not applicable.

## Supplementary Materials

The following supporting information can be downloaded at: https://www.mdpi.com/article/10.3390/ijms241310583/s1.
