# Supplementary material for: Antisera Produced Using an E. coli-Expressed SARS-CoV-2 RBD and Complemented with a Minimal Dose of Mammalian-Cell-Expressed S1 Subunit of the Spike Protein Exhibits Improved Neutralization
Source: Int J Mol Sci. 2023 Jun 24;24(13):10583. doi: 10.3390/ijms241310583 (PMC10341788; doi:10.3390/ijms241310583)
Supplement: Supplementary file 1 [file ijms-24-10583-s001.zip › ijms-2383274-supplementary.pdf]

Supplemental Figures

Figure S1

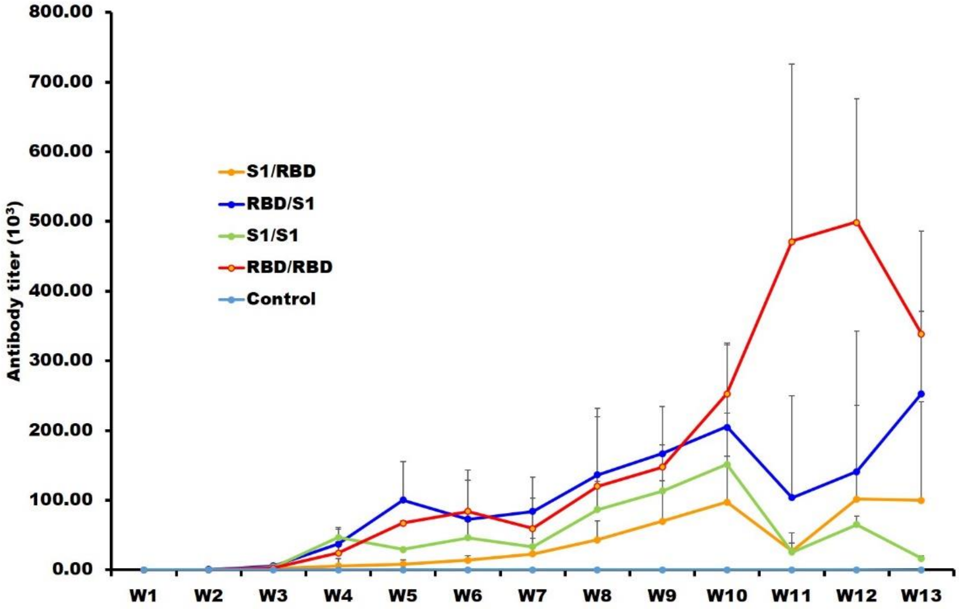

Long-term persistence of SARS-CoV-2-RBD-induced immune response in mice: The average anti-SARS-CoV-2-RBD titers produced by the immunization groups remained high for 13 weeks, indicating a long-term immune response. Anti-SARS-CoV-2 RBD sera (IgG) titer assayed by ELISA: IgG detection by ELISA was performed using the tail bleeding (TB) sera. **W** indicates the tail bleeding week. (**A**) Each line shows the average IgG titer of the mice in each group. The results are indicated as mean ± standard deviation (SD).

Figure S2:

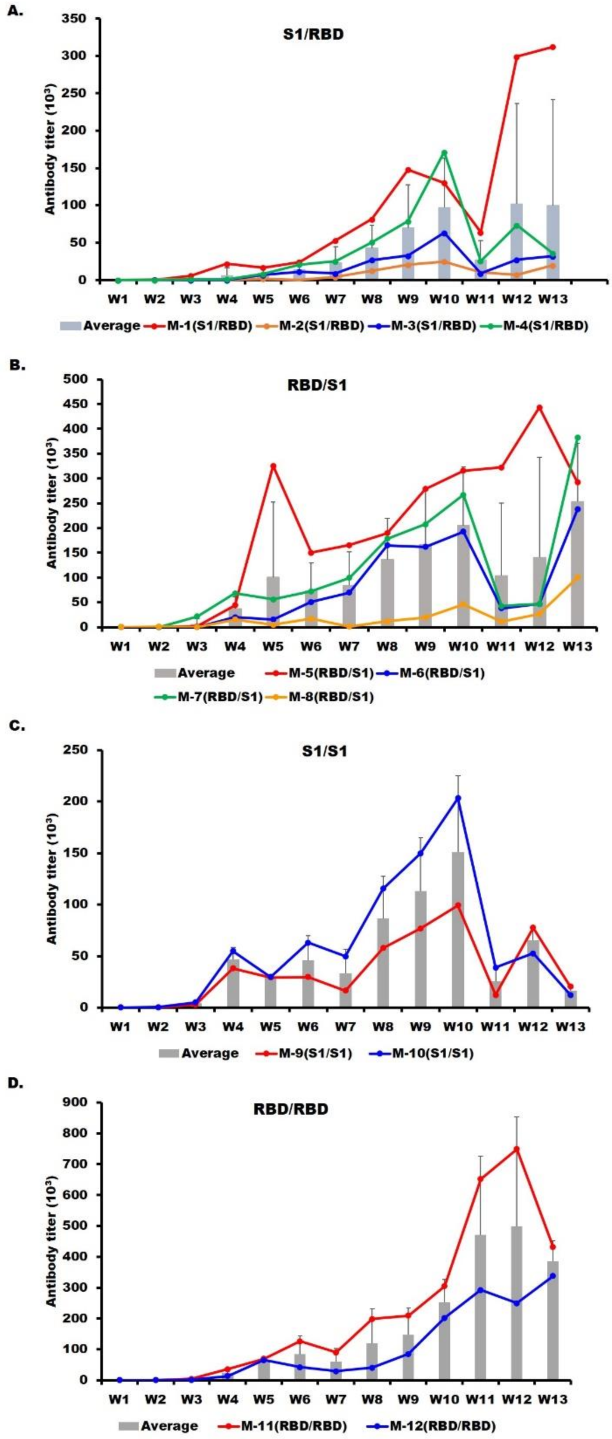

Figure S2: Anti-SARS-CoV-2 RBD sera (IgG) titer assayed by ELISA: IgG detection by ELISA was performed using the tail bleeding (TB) sera from each week for the groups **A.** S1/RBD, **B.** RBD/S1, **C.** S1/S1, **D.** RBD/RBD. **W** indicates the tail bleeding week. Each circle indicates an individual mouse's IgG titer and the bars show the average titer. The results are indicated as mean ± standard deviation (SD).

Table: S1

The average IgG antibody titer was assessed using the 10th week tail bleeding anti sera (the same sera is used for performing the pseudovirus neutralization assay).

	**S1/RBD**	**RBD/S1**	**S1/S1**	**RBD/RBD**
Week 10<br>Average antibody titer	97323.57	205266.88	151125.63	253204.41

Table: S2

Percentage of neutralization assessed using Sars-CoV-2 D614G Pseudovirus with the 10th week tail bleeding anti sera.

**ID**	**Immunisation Group**	**% Neutralisation**
Mice1	S1/RBD	69.44
Mice2	S1/RBD	81.49
Mice3	S1/RBD	85.25
Mice4	S1/RBD	77.92
Mice5	RBD/S1	83.08
Mice6	RBD/S1	83.33
Mice7	RBD/S1	78.45
Mice8	RBD/S1	78.86
Mice9	S1/S1	48.12
Mice10	S1/S1	64.55
Mice11	RBD/RBD	50.43
Mice12	RBD/RBD	32.68
